# Quantitative Organization of GABAergic Synapses in the Molecular Layer of the Mouse Cerebellar Cortex

**DOI:** 10.1371/journal.pone.0012119

**Published:** 2010-08-12

**Authors:** Federica Briatore, Annarita Patrizi, Laura Viltono, Marco Sassoè-Pognetto, Peer Wulff

**Affiliations:** 1 Department of Anatomy, Pharmacology and Forensic Medicine, University of Turin, and National Institute of Neuroscience-Italy, Torino, Italy; 2 Institute of Medical Sciences, Foresterhill, University of Aberdeen, Aberdeen, United Kingdom; The Research Center of Neurobiology-Neurophysiology of Marseille, France

## Abstract

In the cerebellar cortex, interneurons of the molecular layer (stellate and basket cells) provide GABAergic input to Purkinje cells, as well as to each other and possibly to other interneurons. GABAergic inhibition in the molecular layer has mainly been investigated at the interneuron to Purkinje cell synapse. In this study, we used complementary subtractive strategies to quantitatively assess the ratio of GABAergic synapses on Purkinje cell dendrites versus those on interneurons. We generated a mouse model in which the GABA_A_ receptor α1 subunit (GABA_A_Rα1) was selectively removed from Purkinje cells using the Cre/loxP system. Deletion of the α1 subunit resulted in a complete loss of GABA_A_R aggregates from Purkinje cells, allowing us to determine the density of GABA_A_R clusters in interneurons. In a complementary approach, we determined the density of GABA synapses impinging on Purkinje cells using α-dystroglycan as a specific marker of inhibitory postsynaptic sites. Combining these inverse approaches, we found that synapses received by interneurons represent approximately 40% of all GABAergic synapses in the molecular layer. Notably, this proportion was stable during postnatal development, indicating synchronized synaptogenesis. Based on the pure quantity of GABAergic synapses onto interneurons, we propose that mutual inhibition must play an important, yet largely neglected, computational role in the cerebellar cortex.

## Introduction

The cerebellar cortex is one of the most regular and best characterized structures in the mammalian brain [1]–[3]. Its laminated structure, formed by a relatively small number of neuronal types, and its delayed postnatal development, have greatly facilitated experimental analyses aimed at understanding the function and developmental assembly of neuronal networks [4]–[11]. However, our comprehension of cerebellar microcircuits is far from complete. In fact, although excitatory input pathways have been investigated in detail [12], much less is known about the organization of local circuits mediated by inhibitory interneurons.

In this study, we investigated inhibitory synaptic circuits in the molecular layer (ML). Stellate and basket cells are the only ML interneurons (MLIs) known to use GABA as a neurotransmitter [13]. They are distinguished by their position in the upper and lower ML and by their axonal distribution [1], [3], although intermediate forms have been described, raising the possibility that MLIs represent a continuum that varies gradually [14], [15]. Basket cell axons, in particular, surround the cell bodies of Purkinje cells and also form a characteristic plexus around the axon initial segment, whereas stellate cells make synapses exclusively on the dendritic arbor. Collectively, MLIs provide feed-forward and lateral inhibition to Purkinje cells, thus controlling their firing rate, the precise timing of action potential firing and the spread of activity [4], [16], [17]. In addition to targeting Purkinje cells, MLIs make synapses with each other, and likely with Golgi cell dendrites. The existence of such synapses is supported by both electron microscopic analyses [3] and electrophysiological recordings [16], [18]–[20]. However, mutual inhibition between interneurons is largely neglected in theoretical considerations of cerebellar circuit function, based on the assumption that Purkinje cells receive most of the inhibitory synapses in the ML [5], [6], [21]–[25].

GABA_A_ receptors (GABA_A_Rs) are heteropentameric chloride channels assembled from a large family of homologous subunits [26], [27]. Although 13 different subunits have been found in cerebellum [28], only a limited repertoire of receptor subtypes is present in the ML, where the α1βxγ2 subunit combination (with βx indicating one of the three β subunit variants) is by far the most abundant [28], [29]. Receptors containing the α1 subunit have been found in Purkinje cells and ML interneurons, but not in Golgi cells [30], [31]. Notably, GABA_A_Rα1 is the only α subunit expressed in mature Purkinje cells, and deletion of this subunit results in a complete loss of synaptic GABA_A_Rs [32], [33]. α3βxγ2 receptors are also present in the ML. They account for ∼8% of total GABA_A_R clusters in the ML [33], [34] and appear to be expressed predominantly by Golgi cells [35].

The goal of the present study was to provide an accurate estimate of the proportion of GABAergic synapses onto Purkinje cells *versus* those onto interneurons in the ML of the mouse cerebellum. We used two complementary approaches: 1, we generated conditional knockout mice in which GABA_A_Rs were selectively removed from PCs by deletion of the α1 subunit and analysed the density of residual GABA_A_R clusters in interneurons; 2, we used antibodies against α-dystroglycan to label selectively GABAergic synapses on Purkinje cells [33]. The results indicate that synapses between interneurons account for a large proportion of GABAergic synapses in the ML.

## Materials and Methods

All procedures involving experimental mice were approved by the Italian Ministry of Health and by local authorities in accordance with national (Legislative Decree 116/92 and law n. 413/1993) and international (Directive 86/609/EEC and the recommendation 2007/526/EC from European community) laws and policies.

### Generation of PC-Δα1 mice

Mice homozygous for a conditional GABA_A_Rα1 gene (α1lx; exon 9 flanked by loxP sites; [36]) were crossed with mice heterozygous for α1lx and hemizygous for an L7Cre transgene [37]. Littermates of the following genotypes were used for the experiments: α1lx/α1lx/L7Cre (PC-Δα1) and α1lx/α1lx (littermate controls). Mice were genotyped by PCR analysis of genomic DNA from biopsies using the following primer pairs:

α1lx5′_s (5′-CAGCTCTATAAATATCTCTGAGTACC-3′) plus

α1lx5′ _as (5′-GATTGTGATGGTGGAGTCAGAATATG-3′)

to test for the α1lx allele (240 bp band for WT, 280 bp band for α1lx), and:

Cre1 (5′-GACCAGGTTCGTTCACTCATGG-3′) plus

Cre2 (5′-AGGCTAAGTGCCTTCTCTACAC-3′)

to test for the Cre recombinase transgene (250 bp band for L7Cre).

For quantification of GABAergic synapses using α-dystroglycan immunoreactivity, experiments were also performed on WT mice (C57BL/6 stain). For developmental analyses, mouse pups were taken at different postnatal days, defining the day of birth as P0.

### Antibody characterization

The primary antibodies used in the present study are listed in the Supplementary Table S1. Polyclonal antibodies directed against GABA_A_R subunits (α1, α3, γ2) were kindly provided by Dr. Jean-Marc Fritschy (University of Zurich, Zurich, Switzerland). They were raised in rabbits (α1 subunit) and guinea pigs (α1, α3 and γ2) using synthetic peptides derived from the corresponding cDNAs and coupled to KLH. Each of these antibodies labels a single band in Western blots of crude brain membrane fractions (α1: 50 kDa; α3: 59–60 kDa; γ2: 43–48 kDa), and labelling is abolished by competition with the respective antigens [38], [39]. Moreover, immunolabelling specificity has been verified in brain sections of knockout mice lacking the corresponding GABA_A_R subunit [35], [40], [41].

An antiserum against the synaptic adhesion molecule neuroligin-2 (NL2) was kindly provided by Dr. Frédérique Varoqueaux (Max-Planck Institute of Experimental Medicine, Göttingen, Germany). This antibody has been raised in rabbits against a C-terminal sequence corresponding to residues 750–767 of rat NL2, coupled to KLH, and recognizes a single band of 105 kDa in Western blots of rat and mouse brain homogenates. Immunolabelling is abolished by preabsorption with the peptide antigen, and no bands are visible in Western blots of NL2 knockout mouse brain homogenates. Moreover, the antiserum recognizes NL2, but not NL1 or NL3, in transfected cells [42]. We also used a commercially available antiserum (Synaptic Systems, Göttingen; catalog No. 129 203; generous gift of Dr. Henrik Martens), which was raised in rabbits against the same sequence and affinity purified with the immunogen. Both antisera produced similar labelling patterns in mouse brain sections processed for immunofluorescence as described below.

The monoclonal antibody against α-dystroglycan (clone VIA4-1) was obtained from Upstate cell signaling solutions (Lake Placid, NY). This antibody was raised against rabbit skeletal muscle membrane preparations and recognizes a single band of approximately 156 kDa in Western blots of skeletal muscle lysate [43]. In neurons, mAbVIA4-1 gives a punctuate labelling that colocalizes with GABA_A_R at postsynaptic specializations and is abolished by genetic deletion of dystroglycan [33], [44].

To localize GABA in postembedding experiments, we used an affinity-purified antibody raised in rabbits against GABA conjugated to BSA (Sigma-Aldrich, St. Louis, MO; catalog No. A 2052). This antibody showed positive binding to GABA and GABA-KLH, but not to BSA, in a dot blot assay. Furthermore, the antibody has been extensively characterized by immunogold investigations, where it gives strong labelling of GABAergic presynaptic profiles making symmetric synapses [32].

Antibodies against carbonic anhydrase 8 (Car8), a selective marker of Purkinje cells [33], were produced in rabbit and guinea pig against residues 33–61 of mouse Car8. These antibodies recognize a single band of 35 kDa in mouse cerebellar homogenates. Moreover when applied to immunofluorescence on parasagittal brain sections, the antibodies strongly label Purkinje cells, and the immunoreactivity is abolished by preabsorption with the immunogen [33].

Mouse monoclonal anti-calbindin (Swant, Bellinzona, Switzerland; code No. 300) was raised against calbindin D-28k purified from chicken gut. This antibody reacts specifically with calbindin (28 kDa) in immunoblots of brain homogenates of different species, and does not cross-react with calretinin or other known calcium-binding proteins [45]. No labelling is visible in brain sections obtained from calbindin D-28k knockout mice [46]. Rabbit anti-parvalbumin (Immunostar, Stillwater, MN; catalog No. 24428) was raised against parvalbumin purified from rat muscle. This antiserum has been characterized extensively by immunohistochemistry. In sections of the cerebellar cortex, it labels selectively neurons that are known to contain parvalbumin, i.e. Purkinje cells and ML interneurons. In addition, double labelling with other well-characterized monoclonal antibodies against parvalbumin results in precise colocalization (data not shown).

### Immunofluorescence and confocal microscopy

In most cases we used a brief-fixation protocol that has been optimized for *in situ* detection of postsynaptic molecules [34]. Briefly, the cerebellar vermis was cut manually in sagittal slices (∼1 mm) that were fixed by immersion in 4% formaldehyde for 30 minutes (for details, see ref. [47]). The sections were then cryoprotected in sucrose (10%, 20% and 30%), and sectioned with a cryostat. For detection of calbindin and parvalbumin, mice were perfused with 4% formaldehyde, and their cerebellum was postfixed in the same fixative solution for 4 hours. After cryoprotection, the cerebellum was cut with a cryostat into sagittal sections that were collected on gelatin-coated slides.

For immunofluorescence, the sections were first blocked with normal goat (or donkey) serum (3% in phosphate buffered saline, PBS), and then incubated overnight with combinations of two or three primary antibodies raised in different species [34]. After rinsing in PBS, the sections were incubated with the appropriate secondary antibodies, raised either in goat or in donkey and conjugated to one of the following fluorophores: Alexa 488, Alexa 568 (Molecular Probes, Eugene, Oregon), or the cyanine-derived Cy3 and Cy5 (Jackson Immunoresearch, West Grove, PA). Finally, the sections were rinsed and coverslipped with Dako fluorescence mounting medium (Dako Italia, Italy).

Confocal images were acquired with a laser scanning confocal microscope (Zeiss LSM5 Pascal), using the multi-track mode. Quantification of postsynaptic clusters was done in confocal images (512×512 pixels) acquired with a ×100 oil-immersion objective (1.4 NA) at a magnification of 8.1×10^−3^ µm^2^/pixel and the pinhole set at 1 Airy unit. Clusters were quantified manually or with the NIH Image J program, as described previously [34]. Colocalization between two different molecules was analyzed in segmented images with the Imaris software (release 4.2; Bitplane, Zurich, Switzerland; for details, see ref. [34]).

The numerical density of postsynaptic GABA_A_R clusters was estimated using the disector method [48] applied to z-stacks of confocal sections spaced 0.2 µm. We first counted all clusters contained within an individual volume but not touching the exclusion sides (top and right side of each optical section). Clusters were counted in a 3D region of neuropil comprised between the fourth and fourteenth section (edge planes) of a stack of 15 sections, using the sections immediately above or below to facilitate the identification of clusters intersecting the edge planes. Both edge planes were defined alternatively as inclusion or exclusion planes, thus the number of clusters intersecting both edge planes was divided by half and the resulting value was subtracted from the total number of clusters. This was done to minimize differences in labelling intensity due to uneven penetration of the antibodies throughout the reconstructed volume. Finally, the number of clusters was divided by the volume of the neuropil examined, resulting in synapse density.

### Electron microscopy

Mice were perfused with a fixative containing 1% formaldehyde and 1% glutaraldehyde in PB. The cerebellum was dissected, postfixed in the same fixative overnight, and the vermis was cut into sagittal sections with a scalpel. The sections were postfixed in osmium tetroxide (1% in 0.1 M cacodylate buffer), dehydrated in ethanol and embedded in Epon-Araldite. Ultrathin sections were collected on nickel mesh grids and processed for immunogold labelling for GABA as described in ref. [49], using as secondary antibodies goat Fab fragments coupled to 10 nm gold particles (British BioCell International, Cardiff, UK). Sections were analyzed with a JEM-1010 electron microscope (Jeol, Japan) equipped with a side-mounted CCD camera (Mega View III, Olympus Soft Imaging Solutions, Germany).

## Results

### Purkinje cell-selective ablation of GABA_A_ receptors

**Figure 1:**
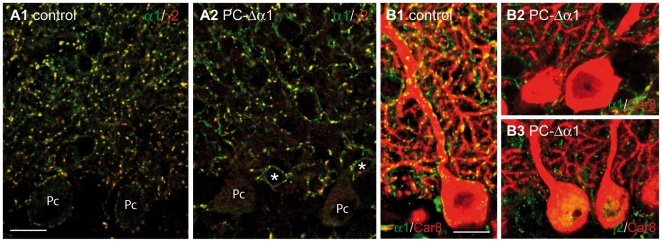
Loss of GABA_A_Rs from Purkinje cells of PC-Δα1 mice. (**A1,A2**) Confocal images showing the distribution of GABA_A_Rα1 (green) and GABA_A_Rγ2 (red) in the cerebellar cortex of control (A1) and PC-Δα1 mice (A2). Note that in both conditions most GABA_A_Rγ2-positive clusters colocalize with the α1 subunit. A few clusters labelled for GABA_A_Rγ2 but not for GABA_A_Rα1 likely represent synapses containing α3-GABA_A_Rs. No surface labelling is visible in Purkinje cells (Pc) of PC-Δα1 mice, whereas MLIs (asterisks) are recognized by extrasynaptic labelling of the α1 subunit (A2). Double-labelled clusters (yellow) identify postsynaptic GABA_A_R aggregates on the cell body and the dendrites of MLIs (A2). (**B1**) Clustered distribution of GABA_A_Rα1 (green) in control Purkinje cells labelled for Car8 (red). (**B2,B3**) GABA_A_R clusters (B2: GABA_A_Rα1; B3: GABA_A_Rγ2) are not visible in Purkinje cells of PC-Δα1 mice. Scale bars: 15 µm.

To determine the contribution of interneuron-interneuron connections to the total number of GABAergic synapses in the ML, we selectively removed the α1 subunit and thus GABA_A_Rs from Purkinje cells using the Cre/loxP system (see Materials and Methods). PC-Δα1 mice appeared healthy and showed no obvious neurological abnormalities (data not shown). We analyzed the organization of inhibitory synapses in adult (>P35) PC-Δα1 and control mice, using antibodies against GABA_A_RΔα1 and GABA_A_Rγ2. In control animals, these subunits colocalized in the large majority of GABAergic synapses in the ML, and clearly outlined the cell body and major dendritic segments of Purkinje cells (Fig. 1A1,B1). Only a few puncta were positive for GABA_A_Rγ2 but not GABA_A_Rα1. Such puncta were not associated with Purkinje cells and likely represent synapses onto Golgi cells expressing the α3 subunit. In contrast, in PC-Δα1 mice no punctuate immunolabelling for either GABA_A_Rα1 or GABA_A_Rγ2 was visible in Purkinje cells (Fig. 1A2), as also shown by double labelling for the Purkinje cell-specific Car8 (Fig. 1B2,B3), indicating that ablation of the α1 subunit resulted in a complete loss of postsynaptic GABA_A_R aggregates. However, numerous GABA_A_R clusters were visible in the neuropil, demonstrating the presence of inhibitory synapses onto interneurons (Fig. 1A2,B2,B3).

**Figure 2:**
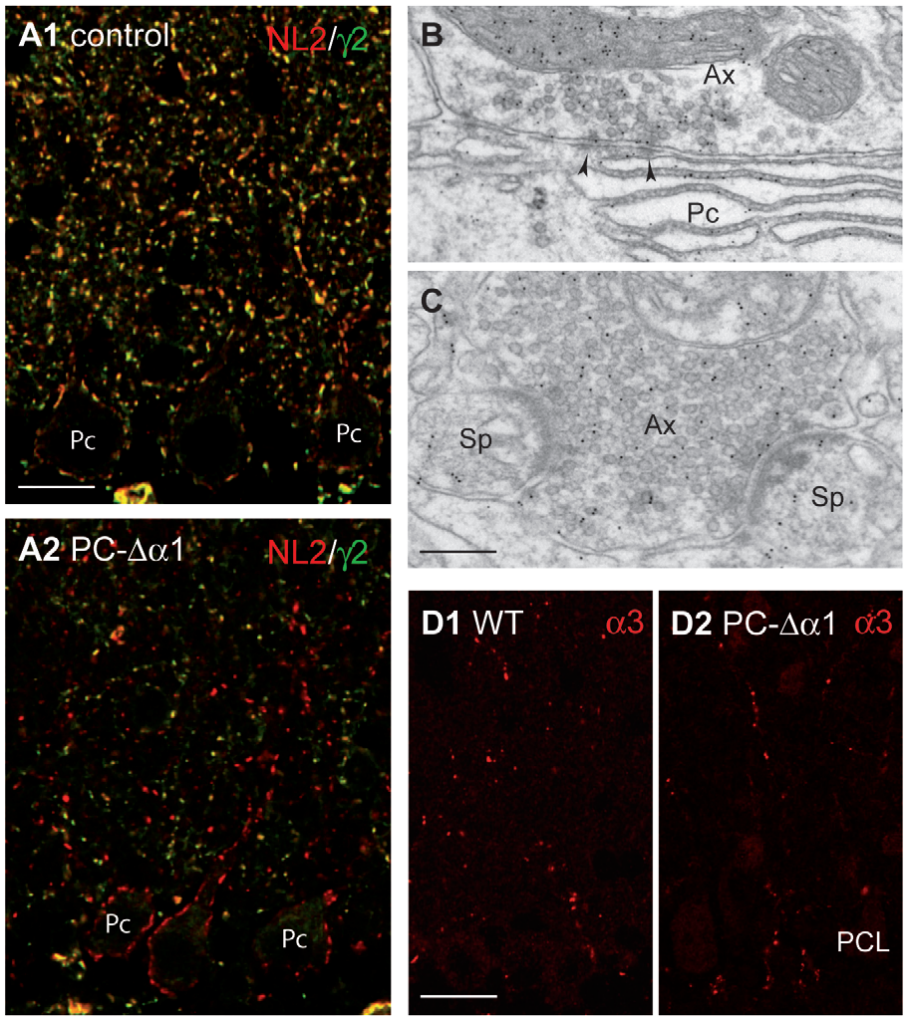
Synaptic organization in the cerebellum of PC-Δα1 mice. (**A1,A2**) Double labelling for GABA_A_Rγ2 (green) and NL2 (red) in control (A1) and PC-Δα1 mice (A2). NL2 colocalizes extensively with the γ2 subunit and also clusters at postsynaptic sites lacking GABA_A_Rs in Purkinje cells of PC-Δα1 mice. (**B,C**) Electron micrographs of the ML of a PC-Δα1 mouse showing that GABA-immunopositive axon terminals (Ax) make both conventional, symmetric synapses (B, arrowheads) with Purkinje cell dendrites (Pc) and heterologous synapses (C) with spines (Sp). (**D1,D2**) Similar distribution of α3-GABA_A_Rs in the ML of control (D1) and PC-Δα1 mice (D2). PCL, Purkinje cell layer. Scale bars: A = 15 µm. B,C = 200 nm. D = 20 µm.

To further validate the efficacy of the Cre-mediated deletion of GABA_A_Rs, we performed double labelling for GABA_A_Rγ2 and the synaptic adhesion molecule NL2, that clusters at inhibitory synapses independently of GABA_A_Rs [33]. Indeed, Purkinje cells of PC-Δα1 mice were decorated by numerous NL2 clusters, that were unlabelled for GABA_A_Rs (Fig. 2A2). However, the density of NL2 clusters in both the internal and external parts of the ML was significantly reduced compared to control animals (iML: mean ± SEM/1.000 µm^2^ = 88.2±2.6 in control and 69.2±1.3 in PC-Δα1, n = 3; P<0.0001, unpaired *t*-test; eML: mean ± SEM/1.000 µm^2^ = 86.8±3.6 in control and 52.6±1.2 in PC-Δα1, n = 3; P<0.0001, unpaired *t*-test), suggesting a reduced density of inhibitory synapses. Using electron microscopy, we confirmed that GABA-immunopositive axons made symmetric synapses with the cell body and the dendrites of mutant Purkinje cells (Fig. 2B). We also found many heterologous contacts between GABA-immunopositive boutons and dendritic spines containing asymmetric synaptic specializations (Fig. 2C). Together, these data reveal similarities in the synaptic organization of PC-Δα1 mice and global GABA_A_Rα1 KO mice [32], [33], and indicate that in both mouse models the selective loss of GABA_A_Rs from Purkinje cells reduces GABAergic innervation and causes the appearance of heterologous synapses with spines.

Ablation of the α1 subunit in global GABA_A_Rα1 KO mice triggers a dramatic increase in the density of postsynaptic sites expressing GABA_A_Rα3 in the ML, which has been interpreted as a reorganization of cerebellar networks [35]. In contrast, the density of GABA_A_Rα3-immunopositive clusters (Fig. 2D1,D2) was comparable in control (mean ± SEM/1000 µm^2^ = 7.2±0.6, representing ∼8% of the total population of GABA_A_R aggregates) and PC-Δα1 mice (6.2±0.5, n = 3; p>0.1, unpaired *t*-test), revealing that the selective loss of GABA_A_Rs from Purkinje cells did not affect the expression of GABA_A_R subunits in cerebellar interneurons (see Discussion). In a separate set of experiments with antibodies against calbindin and parvalbumin, we did not find any obvious defect in the number of ML interneurons (mean ± SEM/10.000 µm^2^ = 9.4±0.1 in control and 9.6±0.4 in PC-Δα1, n = 3; P>0.1, unpaired *t*-test) and Purkinje cells (mean ± SEM/100 µm = 4.1±0.09 in control and 4±0.1 in PC-Δα1; P>0.1, unpaired *t*-test), as well as in the organization of parallel fibers and climbing fibers (data not shown). Therefore, apart from the reduced GABAergic innervation of Purkinje cells and the presence of heterologous synapses, there were no obvious alterations of GABAergic circuits in the ML of PC-Δα1 mice.

### Ablation of the α1 subunit does not occur synchronously in Purkinje cells

**Figure 3:**
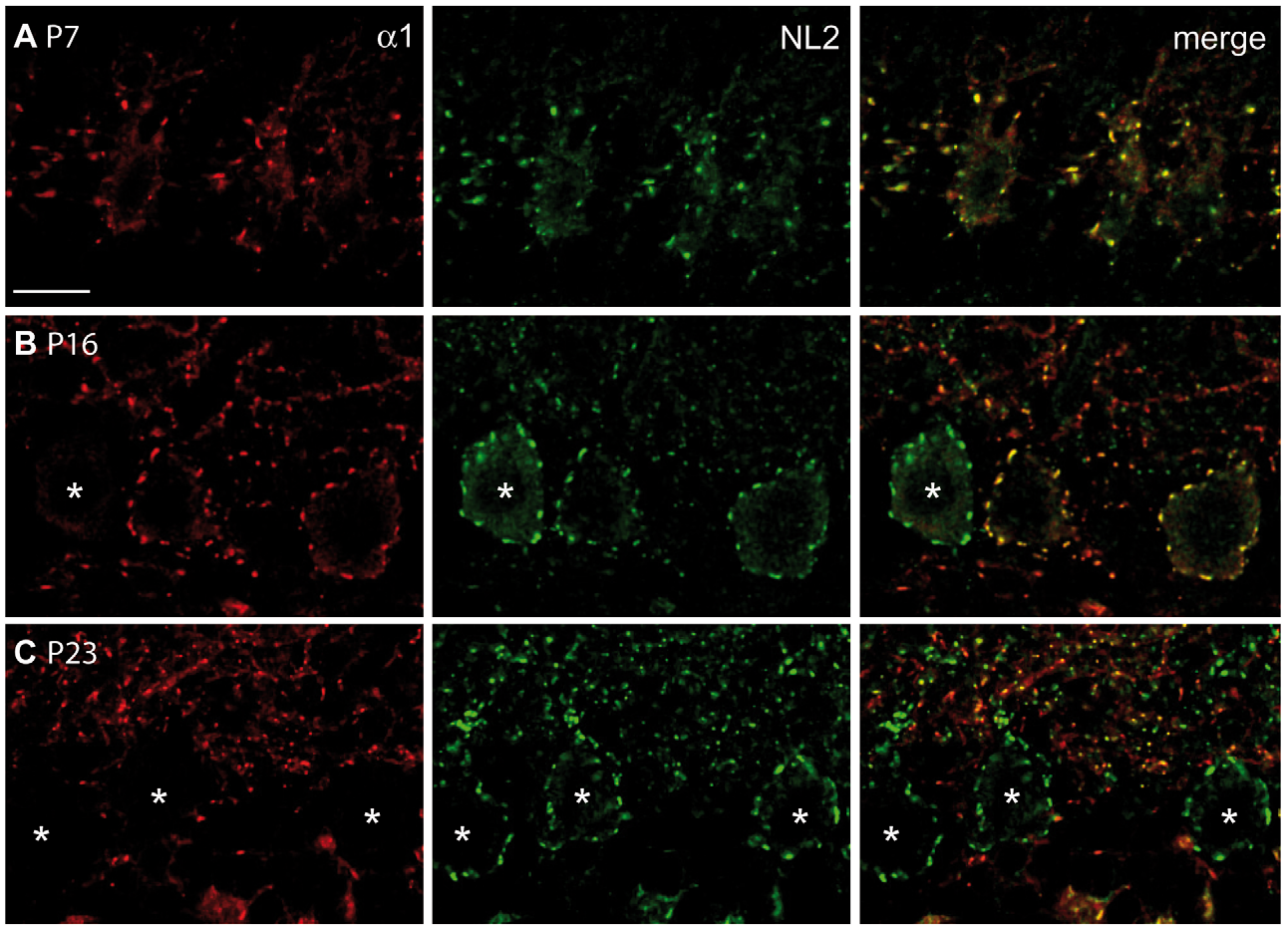
Ablation of GABA_A_Rs is protracted during postnatal development and is asynchronous among different Purkinje cells. Double labelling for GABA_A_Rα1 (red) and NL2 (green) in the cerebellar cortex of P7 (**A**), P16 (**B**) and P23 (**C**) PC-Δα1 mice. All P7 Purkinje cells express postsynaptic GABA_A_R clusters, colocalized with NL2 (A). Purkinje cells labelled for NL2 but not GABA_A_Rs (asterisks) are visible at P16 (B). By P23, practically all Purkinje cells are immunonegative for GABA_A_Rs (**C**). Scale bar: 20 µm.

To map the time window in which GABA_A_R loss occurs in Purkinje cells, we performed a developmental analysis. We found that GABA_A_Rα1 immunoreactivity was gradually lost during the second and third postnatal weeks. Thus, in P7 mice virtually all Purkinje cells were immunolabelled for GABA_A_Rα1 (Fig. 3A), whereas at later stages (P14–P16) labelling of Purkinje cells had a mosaic-like pattern, characterized by immunopositive and immunonegative cells, which were often located side-by-side (Fig. 3B). By P23, essentially all Purkinje cells had lost immunoreactivity for GABA_A_Rs, as determined by visual inspection of their cell bodies (Fig. 3C). A quantitative evaluation of the sequential GABA_A_Rα1 loss was performed in lobule V by comparing immunolabelling for GABA_A_Rα1 with that of NL2 at perisomatic synapses of Purkinje cells. At P7, all NL2-positive Purkinje cells (n = 30 cells) were also labelled for GABA_A_Rα1. The percentage of Purkinje cells expressing both NL2 clusters and GABA_A_Rα1 dropped to 48.3% at P16 (14 out of 29 cells), and no Purkinje cell was found labelled for the α1 subunit at P23 (n = 27 cells). This sequence was accompanied by a progressive increase in the density of “silent” synapses expressing NL2 but no GABA_A_Rs in the ML (Fig. 3B,C). The temporal profile of GABA_A_Rα1 removal is in good agreement with the protracted appearance of Cre recombinase activity in Purkinje cells of the original L7Cre transgenic mouse line (see ref. [37]).

### Quantification of GABAergic synapses between interneurons in PC-Δα1 mice

We next calculated the density of residual GABA_A_R clusters in the ML of adult PC-Δα1 mice, using immunolabelling for GABA_A_Rγ2. This subunit is present in all postsynaptic GABA_A_Rs and thus accounts for both GABA_A_Rα1 and GABA_A_Rα3-positive synapses [29], [34]. Because the density of inhibitory synapses along Purkinje cell dendrites is different in the iML and eML [33], we performed our analysis in confocal fields (46.1×46.1 µm^2^) just above the Purkinje cell layer or below the pial surface. All experiments were on vermal lobule V, but qualitative observations suggested limited variability among different lobules.

In PC-Δα1 mice, the density of GABA_A_Rγ2-immunopositive clusters was reduced to ∼40% in both the iML (mean ± SEM/1.000 µm^2^ = 85.7±1.9 in control and 33.9±0.8 in PC-Δα1, n = 3; P<0.0001, unpaired *t*-test) and eML (mean ± SEM/1.000 µm^2^ = 85.1±4.4 in control and 33.2±1.2 in PC-Δα1, n = 3; P<0.0001, unpaired *t*-test). Therefore, by comparing GABA_A_R cluster density in PC-Δα1 and control mice, we infer that approximately 60% of ML GABAergic synapses are on Purkinje cell dendrites.

### Quantification of GABAergic synapses onto Purkinje cells using α-dystroglycan immunoreactivity

**Figure 4:**
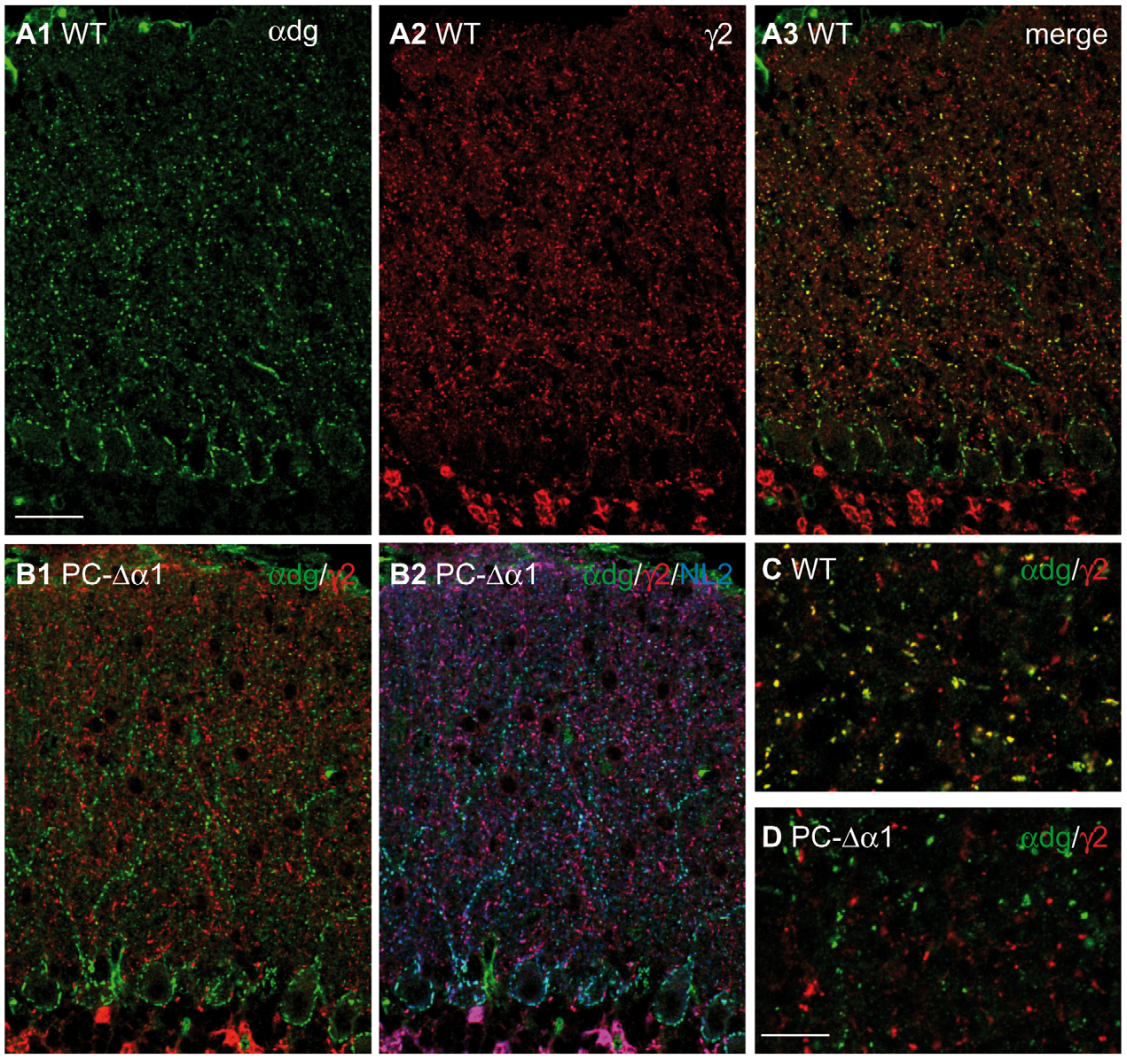
Dystroglycan is present at GABAergic synapses in Purkinje cells but not in cerebellar interneurons. (**A1–A3**) Double labelling for α-dystroglycan (green) and GABA_A_Rγ2 (red) in the cerebellar cortex of a WT mouse. Labelling for α-dystroglycan outlines the cell bodies and major dendrites of Purkinje cells and colocalizes precisely with GABA_A_Rγ2. Labelling for the γ2 subunit is weaker at perisomatic synapses, scarcely visible in these low magnification images. (**B1,B2**) Triple labelling for α-dystroglycan (green), GABA_A_Rγ2 (red) and NL2 (blue) in the cerebellar cortex of a PC-Δα1 mouse. Dystroglycan colocalizes with NL2 exclusively at silent synapses that lack GABA_A_Rs (B2, cyan). NL2 associates with GABA_A_Rs at interneuron-interneuron synapses, where immunolabelling for α-dystroglycan is not visible (B2, magenta). (**C,D**) High-magnification images of the ML showing that in WT mice a subset of GABAergic synapses contain α-dystroglycan (yellow clusters), whereas in PC-Δα1 mice α-dystroglycan never colocalizes with GABA_A_Rγ2-positive clusters. Scale bars: A,B = 30 µm. C,D = 10 µm.

Although many Purkinje cells retain GABA_A_Rs during an extended period of postnatal development (Fig. 3), we cannot exclude compensatory changes in PC-Δα1 cerebellar circuits that could alter the number of inhibitory synapses. We therefore used an alternative approach to estimate the relative amount of GABAergic synapses onto Purkinje cells and interneurons. Purkinje cells are the only cerebellar neurons that express dystrophin and dystroglycan at inhibitory postsynaptic specializations [33], [50], [51]. Notably, in GABA_A_Rα1 KO mice these molecules colocalize with NL2 at silent synapses lacking GABA_A_Rs [33]. Similarly, in PC-Δα1 mice α-dystroglycan was found exclusively at NL2-positive synapses without GABA_A_Rs, confirming that it does not occur at synapses on interneurons (Fig. 4B,D). Moreover, all NL2-positive/GABA_A_R-negative synapses were positive for α-dystroglycan (Fig. 4B2), suggesting that this molecule is a reliable marker of GABAergic synapses on Purkinje cells.

Accordingly, we used double-immunofluorescence for α-dystroglycan and GABA_A_Rγ2 in WT cerebellar sections (Fig. 4A,C) to calculate the ratio of dystroglycan-positive synapses over the total density of GABAergic synapses. In the iML, dystroglycan-positive synapses accounted for 62.8% of the total population of GABAergic synapses (Table 1). This value is almost identical to the percentage of Purkinje cell synapses estimated by comparing PC-Δα1 and control mice. In the eML, we found a lower density of dystroglycan-positive puncta, accounting for 46.5% of GABAergic synapses. This value is even lower than the previous estimate from PC-Δα1 mice (see Discussion). These two sets of experiments reveal that more than one third of GABAergic synapses in the ML occur between interneurons.

**Table 1:** Evaluation of GABAergic synapses on Purkinje cell dendrites by immunolabelling with antibodies against α-dystroglycan.

	GABA_A_Rγ2	α-dystroglycan
eML	84±1.3	39±2.7 (46.5%)
iML	91.5±2.8	57.5±1.8 (62.8%)

Density values are means ± s.e.m. of four different mice and are expressed as number of puncta per 1000 µm^2^. The percentage of synapses immunolabelled for both GABA_A_Rγ2 and α-dystroglycan is given in parenthesis.

**Figure 5:**
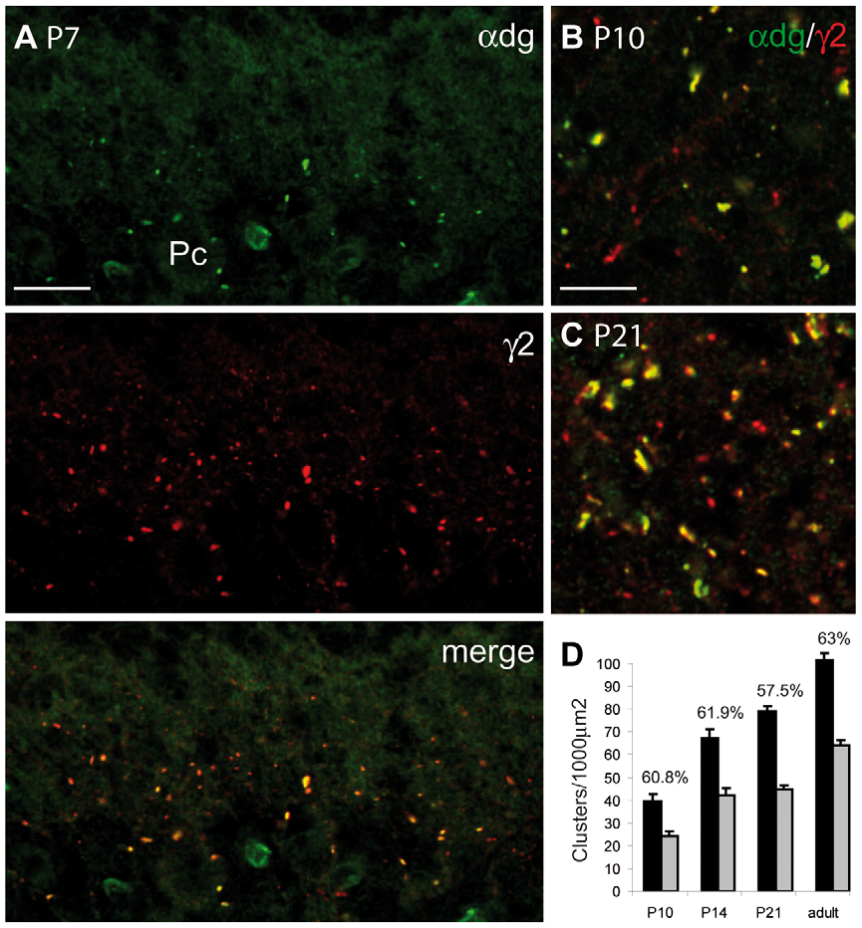
Early synaptic localization of α-dystroglycan and postnatal development of GABAergic synapses in the ML. (**A**) Double labelling for α-dystroglycan and GABA_A_Rγ2 in the cerebellar cortex of a P7 wild-type mouse showing that α-dystroglycan clusters at developing GABAergic synapses onto Purkinje cells (Pc). (**B,C**) Representative images showing that α-dystroglycan associates with a subset of GABA_A_Rγ2-positive synapses in the ML of P10 (B) and P21 (C) mice. (**D**) Synapses were quantified in the iML by counting clusters immunopositive for GABA_A_Rγ2 (black bars) and α-dystroglycan (grey bars). Percentage values express the ratio of α-dystroglycan-positive clusters over the entire population of GABA synapses labelled for GABA_A_Rγ2 (n = 3 mice for each developmental stage). Note that the values vary little during postnatal development. Scale bars: A = 15 µm. B,C = 6 µm.

We then analyzed how stable the ratio of GABAergic synapses is during postnatal development. We found that α-dystroglycan concentrates at postsynaptic sites at early postnatal stages. In fact, practically all perisomatic synapses of Purkinje cells were labelled for this molecule already at P7 (Fig. 5A), and this labelling became more robust at P10. Figure 5 shows examples of double labelling for α-dystroglycan and GABA_A_Rγ2 in cerebellar sections taken at different postnatal stages. Immunoreactivity for these molecules was punctuate, and based on previous analyses [33], [34] we assumed that the large majority of such puncta represented postsynaptic aggregates. Synapse density was calculated in the iML, because this part of the ML was already present at P10 and could be followed throughout development. We found that the density of GABAergic synapses (immunopositive for GABA_A_Rγ2) increased gradually from P10 to adult, as previously reported [33]. Notably, dystroglycan-positive synapses accounted for approximately 60% of total GABAergic synapses at all developmental stages (Fig. 5D). These data confirm previous findings that synaptogenesis in the ML extends beyond the second postnatal week [33], [34]. The constant ratio of inhibitory synapses onto Purkinje cells and interneurons may help to maintain activity patterns and minimize unbalanced activity in the developing cerebellum.

### Unbiased estimate of GABAergic synapse density in the molecular layer

We finally aimed to determine the density (number per unit volume) of GABAergic synapses in the ML. This cannot be accurately estimated from the previous findings, in which fluorescent clusters were counted in individual confocal sections with no exclusion boundaries. While this analysis is effective in determining the ratio of clusters immunopositive for different postsynaptic molecules (e.g. GABA_A_Rγ2 and α-dystroglycan), or the relative abundance of the same population of clusters in the ML of different mouse strains, it may result in a systematic oversampling of larger clusters, given that cluster size (range: 0.4–1.8 µm along the Z axis) exceeds the thickness of confocal sections [48]. We therefore used a stereological approach with an optical disector (see Materials and Methods) to estimate the density of postsynaptic sites immunopositive for the GABA_A_Rγ2 subunit in the iML of wild-type mice. This analysis revealed that postsynaptic GABAergic specializations occur at a density of 104±6.2/1000 µm^3^ (mean ± SEM of four optical disectors). In the same analysis, dystroglycan-immunopositive clusters reached a density of 66.3±5.4/1000 µm^3^, corresponding to 63% of GABAergic synapses impinging onto Purkinje cells.

## Discussion

Understanding neural computation requires detailed knowledge of the intrinsic properties of individual neurons, as well as their connectivity pattern. This can only be achieved by a convergence of anatomical and electrophysiological data. The goal of this study was to acquire quantitative data on the organization of inhibitory synapses in the ML of the mouse cerebellar cortex. While the majority of GABAergic synapses were situated on the dendrites of Purkinje cells, synapses between interneurons occurred at an unexpectedly high density, accounting for approximately 40% of inhibitory synapses in the ML.

The results of our study originate from two distinct sets of experiments. First, we determined the proportion of synapses onto interneurons by specifically eliminating postsynaptic GABA_A_Rs from Purkinje cells in PC-Δα1 mice (Fig. 1). Using this approach we found that synapses between interneurons account for 35–40% of total GABAergic synapses in both the iML and eML. However, previous studies have reported a reorganization of the cerebellar circuitry in GABA_A_Rα1 KO mice [32], [35]. We thus used a second independent approach to confirm our findings: we performed immunohistochemistry in WT mice with antibodies against α-dystroglycan, a Purkinje cell-selective marker of GABAergic synapses (Fig. 4). The results that we obtained with these two complementary approaches were similar. The only notable difference was the lower density of GABAergic synapses onto Purkinje cells in the eML when judged by α-dystroglycan labelling. It is possible that we underestimated dystroglycan-positive synapses in the eML, where synaptic clusters are usually smaller and less intensely labelled. Alternatively, this discrepancy could reflect slight changes in synapse density in the eML of PC-Δα1 mice. This is plausible, as Purkinje cells lose the α1 subunit mostly during the third postnatal week (Fig. 3), largely matching the development of the most superficial part of the ML. Despite these minor differences, and in view of the above considerations, the combined analysis of WT and PC-Δα1 mice indicates that the selective loss of GABA_A_ receptors from Purkinje has little effect on inhibitory synapses between interneurons.

Our findings are based on a sensitive immunofluorescence protocol that allows the detection of individual postsynaptic GABA_A_R clusters with high accuracy [34], [47]. GABA_A_Rs have also been found presynaptically in parallel fiber terminals [52]. However, practically all GABA_A_R clusters visible after immunolabelling with antibodies colocalize with NL2 and gephyrin, which are genuine postsynaptic proteins (Fig. 2; see also refs. [29], [33]). It is likely that presynaptic GABA_A_Rs occur at a low density, as also supported by the weak intensity of immunogold labelling for the α1 subunit [52].

While our findings demonstrate that interneuron-interneuron synapses represent approximately 40% of inhibitory synapses in the ML, the identity of pre- and postsynapic partners remains unclear. All major types of cerebellar interneurons extending their dendrites in the ML (basket, stellate, and Golgi cells) receive inhibitory inputs from other interneurons. However, Golgi cells and MLIs can be distinguished by the differential expression of α1 and α3-GABA_A_Rs. In fact, Golgi cells do not express GABA_A_Rα1 [30], [31], whereas stellate and basket cells express mainly, if not exclusively, GABA_A_Rs with the α1 subunit (ref. [53] and our unpublished observations). As α3-GABA_A_Rs are present in about 8% of GABAergic contacts in the ML (see Results), it follows that synapses onto basket/stellate cells account for ∼30% of GABAergic synapses in the same layer. It should be noted that, while synapses containing α1-GABA_A_Rs are evenly distributed, those containing the α3 subunit have an irregular pattern, consistent with the parasagittal organization of Golgi cell dendrites [54], [55].

Concerning presynaptic structures, it is likely that Purkinje cell recurrent collaterals have only a modest contribution to synapses on interneurons, as their contacts onto basket cells are rare [3]. Lugaro cells make inhibitory synapses with both Golgi [56], [57] and Purkinje cells [58]. In addition, Lugaro cells have been reported to make contacts with stellate/basket cells [59], however numerical estimates of these synapses are not available. While the contribution of synapses made by Lugaro cells remains to be determined, it is likely that the main source of GABAergic synapses onto MLIs are other MLIs, as also supported by electron microscopic analyses [3] and by the high level of connectivity found in electrophysiological studies [19].

An interesting observation of the present study is that the density of GABA_A_Rα3-positive synapses was unchanged in conditional PC-Δα1 mice (Fig. 2D2). This contrasts with the situation in global GABA_A_Rα1 KOs, in which the density of GABA_A_Rα3-positive clusters increased dramatically in the ML [33], [35]. The most likely explanation for this discrepancy is that in global GABA_A_Rα1 KOs the α3 subunit is up-regulated in ML interneurons deprived of the α1 subunit. However, this compensation does not take place in PC-Δα1 mice, where MLIs retain the α1 subunit. If this interpretation is correct, the massive increase in the density of GABA_A_Rα3-positive synapses in global GABA_A_Rα1 KO mice can be explained mainly by a switch in GABA_A_Rα subunit expression in MLIs, rather than a structural reorganization of the cerebellar network as proposed by Kralic et al. (ref. [35]). Interestingly, no compensation by α3-GABA_A_Rs occurs in Purkinje cells of adult GABA_A_Rα1 KO mice, although these neurons express higher-than-normal levels of GABA_A_Rα3 during early stages of postnatal development [33]. Therefore, the genetic program that regulates the expression of GABA_A_Rα3 differs in Purkinje cells and MLIs.

In previous simulations of ML microcircuits, the number of inhibitory synapses made by stellate cells on a Purkinje cell has been estimated at ∼1500 [21]. This value comes from previous studies in rat, according to which the ratio of stellate cells to Purkinje cells is approximately 10∶1 [60], with an individual stellate cell forming on average 149 synapses [14]. This estimation is based on the assumption that the large majority if not all of MLI output synapses are on principal neurons [5]. However, our results indicate that synapses on Purkinje cells account for only ∼60% of ML GABAergic synapses. On the other hand, our study was based on a quantification of postsynaptic clusters representing individual synaptic specializations, whereas in the studies quoted above the number of output synapses of stellate cells was derived from the number of axonal varicosities [14]. Because a single axonal bouton can make multiple synapses, potentially with different neurons, quantification of varicosities may result in an underestimation of the actual number of synapses. In fact, assuming that the dendritic arborization of a Purkinje cell occupies an area of 20000 µm^2^ [61], and that its thickness is 6 µm [6], the volume covered by a Purkinje cell dendrite in the mouse cerebellum should be roughly 120000 µm^3^. Given an estimated density of GABAergic inputs at 66 per 1000 µm^3^, each Purkinje cells should receive approximately 7920 GABAergic synapses. This estimate must be verified after a rigorous calculation of the Purkinje cell dendritic volume in the mouse cerebellum. By extending this reasoning, it should be possible to predict the number of inhibitory synapses received by MLIs. In fact, given the 10∶1 ratio in the number of MLIs and Purkinje cells [60], and assuming that about 60% of GABAergic synapses are on Purkinje cells and about 30% on MLIs (after subtraction of inhibitory synapses onto Golgi cells), a MLI would receive approximately 20 times less GABAergic synapses than a Purkinje cell. Interestingly, this ratio is roughly reflected in the frequency of spontaneous IPSCs recorded in stellate and Purkinje cells [18], [62].

In recent years some progress has been made in understanding the role of stellate and basket cells in cerebellar function. MLIs tightly control the input-output relations of Purkinje cells via feed-forward and lateral inhibition and are required for cerebellum-dependent behaviour and learning [16], [17], [63]–[67]. In this context, the absence of major neurological defects in PC-Δα1 mice is surprising. However, in a highly similar mouse model with a Purkinje cell-selective deletion of GABA_A_Rs (PC-Δγ2 mice with a Purkinje cell-specific deletion of the GABA_A_Rγ2subunit gene), we found a significant decrease in AMPA receptor-mediated current charge transfer after parallel fiber stimulation [17]. This, and possibly other compensatory mechanisms, may help to maintain Purkinje cell excitability in a normal operational range even in the absence of fast synaptic inhibition and account for the absence of gross neurological deficits. On a more subtle level, loss of GABA_A_R-mediated synaptic inhibition in Purkinje cells of PC-Δγ2 mice caused abnormal patterns of simple spikes, strongly compromising cerebellar learning [17].

Our study shows that inhibitory interneurons of the ML are interconnected much stronger than previously anticipated. Such reciprocal connections between inhibitory interneurons are a common motive throughout the CNS from invertebrates to vertebrates [68]. In the cerebellar cortex, reciprocal connections between MLIs have been hypothesized to induce fast (100–250 Hz) oscillations [69], which may aid the encoding of cerebellar information. Alternatively, reciprocal connections between MLIs have been suggested to curtail inhibition during high levels of parallel fiber activity to rapidly return the network to a baseline mode, in which it can process newly arriving parallel fiber inputs [19]. In an extension of this idea, mutual inhibition between interneurons has been proposed to stabilize the firing rate in the interneuron network, as GABAergic transmission between these neurons can be both inhibitory and excitatory depending on the state of the postsynaptic cell. Thus mutual GABAergic innervation may allow the interneuron network to maintain its activity in an optimal operational range to respond to external inputs [20]. Future experiments will have to determine how the strong synaptic coupling between inhibitory interneurons of the ML aids cerebellar information processing.

## Supporting Information

Table S1 (0.05 MB DOC)
