# Supplementary material for: Quantitative Organization of GABAergic Synapses in the Molecular Layer of the Mouse Cerebellar Cortex
Source: PLoS One. 2010 Aug 12;5(8):e12119. doi: 10.1371/journal.pone.0012119 (PMC2920831; doi:10.1371/journal.pone.0012119)
Supplement: Table S1 — (0.05 MB DOC) [file pone.0012119.s001.doc]

### TABLE S1: List of primary antibodies

| **Antibody** | **Immunogen** | **Source – Cat. Number** | **Species** | **Dilution** |
| --- | --- | --- | --- | --- |
| GABA_A_Rα1 | Rat N-terminal peptide 1–16 | H. Mohler and J.-M. Fritschy (Inst. of Pharmacology and Toxicology, University of Zürich, Switzerland) | Guinea pig and Rabbit  polyclonal | 1:5000 |
| GABA_A_Rα3 | Rat N-terminal peptide 1–15 | H. Mohler and J.-M. Fritschy (Inst. of Pharmacology and Toxicology, University of Zürich, Switzerland) | Guinea pig  polyclonal | 1:8000 |
| GABA_A_Rγ2 | Rat N-terminal peptide 1-29 | H. Mohler and J.-M. Fritschy (Inst. of Pharmacology and Toxicology, University of Zürich, Switzerland) | Guinea pig  polyclonal | 1:2000 |
| Neuroligin-2 | Rat C-terminal peptide 750-767 | F. Varoqueaux (Max-Plack Institute of Experimental Medicine, Göttingen, Germany) | Rabbit  polyclonal | 1:2000 |
| Neuroligin-2 | Rat C-terminal peptide 750-767 | Synaptic Systems  (cat. No. 129 203) | Rabbit affinity-purified polyclonal | 1:2000 |
| α-Dystroglycan | Rabbit skeletal muscle membrane preparation | Upstate-Millipore  (cat. No. Q14118 - clone VIA4-1) | Mouse monoclonal | 1:100 |
| GABA | γ-aminobutyric acid (GABA) conjugated to BSA | Sigma-Aldrich  (cat. No. A2052) | Rabbit affinity-purified  polyclonal | 1:2000 |
| Carbonic Anhydrase 8  (Car8) | Peptide 33-61 of mouse carbonic anhydrase 8 | M. Watanabe (University School of Medicine, Sapporo, Japan) | Guinea pig and  Rabbit  polyclonal | 1:500 |
| Calbindin | Calbindin D-28k from chicken gut | Swant  (cat. No. 300) | Mouse monoclonal | 1:10000 |
| Parvalbumin | Parvalbumin purified from rat skeletal muscle | Immunostar  (cat. No. 24428) | Rabbit  polyclonal | 1:3000 |
